# Molecular characterization of hepatitis B virus (HBV) isolated from a pediatric case of acute lymphoid leukemia, with a delayed response to antiviral treatment: a case report

**DOI:** 10.1186/s12887-022-03204-6

**Published:** 2022-03-31

**Authors:** Chien-Yu Chen, Christina Hajinicolaou, Priya Walabh, Luicer Anne Olubayo Ingasia, Ernest Song, Anna Kramvis

**Affiliations:** 1 Hepatitis Virus Diversity Research Unit, Department of Internal Medicine, University of the Witwatersrand, Johannesburg, South Africa; 2 Department of Paediatrics, School of Clinical Medicine, Faculty of Health Sciences, University of the Witwatersrand, Johannesburg, South Africa; 3 Paediatric Gastroenterology, Hepatology and Nutrition Unit, Chris Hani Baragwanath Academic Hospital, Johannesburg, South Africa; 4 Paediatric Gastroentrology, School of Clinical Medicine, Faculty of Health Sciences, University of the Witwatersrand, Johannesburg, South Africa; 5 Department of Internal Medicine, Charlotte Maxeke Johannesburg Academic Hospital, Johannesburg, South Africa

**Keywords:** Acute lymphoblastic leukemia, Case report, Chronic HBV infection, Genotypes, Nucleot(s) ides analogue (NA) treatment, Mutations, Partial virologic response, Viral breakthrough

## Abstract

**Background:**

Tenofovir disoproxil fumarate (TDF) is effectively used as the first-line antiviral for chronic hepatitis B virus (HBV) infection in adults and children older than 12 years. To date, no confirmed case of virologic breakthrough (VBT) in a pediatric case has been reported.

**Case presentation:**

Here we describe a case of a 5-year old, asymptomatically infected with HBV infection two months after chemotherapy for precursor B acute lymphoblastic leukemia (ALL). Although the 5-year old male is South African, his family originated from Guinea. At the end of the one-year follow-up, the infection progressed to chronic HBV infection, with a high viral load. At 36 weeks (8 months) post-treatment with lamivudine (LAM), there was a partial virologic response (PVR) and after 61 weeks (14 months), he was switched to TDF rescue monotherapy. Even with TDF treatment, he still experienced VBT and subsequent PVR. The full-length genome of HBV isolated 78 weeks after the switch to rescue TDF monotherapy was sequenced and belonged to genotype E. In addition to the LAM mutations (rtS256G and rtM267L), missense mutations in B-cell, T-cell, HLA class I and II-restricted epitopes emerged, which were to evade and escape host surveillance, leading to delayed viral clearance, persistence and disease progression. Two further events of VBT occurred between weeks 113 and 141 of TDF rescue-therapy. Viral loads and liver enzymes are normalizing progressively with long-term therapy.

**Conclusion:**

Although the host immune reconstitution may be delayed, prolonged TDF treatment was effective in treating this pediatric case of HBV infection with VBT and PVR.

## Background

HBV infection is an ongoing global health problem. According to the World Health Organization (WHO) global coverage rate of three doses of the hepatitis B virus (HBV) vaccine is estimated to be 84%. This vaccination coverage has resulted in a decrease in the incidence of HBV infection. Nevertheless, HBV infection persists in highly endemic regions, including Africa and South East Asia.

Early childhood HBV infection leads to a much higher rate of persistent infection and long-term complications such as liver cirrhosis and hepatocellular carcinoma (HCC) [1, 2]. In endemic countries, HBV infection occurs mainly during infancy and early childhood. The risk of developing chronic infection is higher in newborns (90%) than in infants/young children (< 5 years old) (25–30%) and immunocompetent older children and adults (< 5%) [3]. Therefore, childhood HBV infection presents medical and public health challenges. In regions with a high prevalence, HBV infection is mostly transmitted either from mother to child at birth (perinatally) as seen in Asia or through exposure to infected blood or body fluid (horizontally) early in life mainly from HBsAg-positive family members/household contacts, playmates or by unsafe medical interventions as seen in Africa [4]. Perinatal or childhood infection is distinct from infection in adulthood [5]. It is characterized by a long-lasting immune-tolerant phase, with no or few symptoms with mild and stable liver disease, persistently high HBV viral load (>10^7^copies/mL), minimal or no inflammation in the liver [6].

Acute lymphoblastic leukemia (ALL) is the most common childhood malignancy, accounting for approximately 25% of all childhood cancers and about 75% of pediatric acute leukemias [7]. A majority of ALL cases develop in children and teenagers (under 15 years of age), mainly in males, with peak incidence in the 2- to 5-year age group. ALL is identified morphologically by more than 25% of leukemic bone marrow blasts [8]. ALL is further subtyped into either B or T cell lineage ALL (WHO classification), being predominately B-cell lineage (85%) [9]. Fortunately, with the improvement in chemotherapy technologies and conservative treatment, the 10-year survival rate for pediatric ALL is 91% [10]. However, infectious complications remain major causes of morbidity and mortality in ALL patients.

Patients with acute leukemia (including ALL) are immunocompromised, characterized by impaired leukocyte proliferation and aberrant maturation, resulting in compromised granulocyte function [11, 12]. In addition, a weaker immune defence system renders pediatric patients more susceptible to bacterial, fungal, and viral infections [11]. Furthermore, medications used in standard chemotherapy regimens are known to be immune-suppressive by affecting leukocyte function, resulting in prolonged neutropenia [12]. Prolonged neutropenia is a risk factor for infections in ALL [13, 14]. Infectious complications are the leading cause of treatment-related morbidity and mortality in pediatric ALL [15] and are highest during the induction and consolidation phases [16, 17].

HBV infection is frequent in patients with acute leukemia [18, 19], and children with this haematological dyscrasia have a higher risk of chronic HBV infection [20], especially for those living in highly endemic countries [21]. For patients with hematological malignancies, new viral infections after the onset of ALL and reactivation of latent infection are the common manifestations of infection during the period of weaker immunity [16, 22]. The general guideline consensus is to routinely screen high-risk patients, especially individuals born in intermediate or high HBV prevalence countries, for HBsAg, anti-HBc, and anti-HBs, prior to immunosuppressive therapy [23].

Here, we report a young male in ALL remission with chronic HBV, genotype E infection. He was treated with lamivudine (LAM), followed by tenofovir (TDF) as rescue monotherapy. Multiple events of virologic breakthrough (VBT) and partial virologic response (PVR) were observed during antiviral treatment, with HBV developing LAM resistance but not TDF resistance mutations. Immune escape mutations in immune epitopes were also detected. These mutations could have induced ineffective HBV-specific immune responses, favoring HBV persistence, leading to VBT and PVR.

## Case presentation

In October 2010, while visiting Angola, the one year old South African-born male, whose parents originated in Guinea, first became ill and was admitted to the hospital and received two blood transfusions. The child arrived in Johannesburg acutely ill. He was profoundly anemic with bruises, in respiratory distress, and congestive cardiac failure. Before admission, the patient was transfused with packed cells, received alkalinizing fluid, and two full days of Amoxicillin and Clavulanate prescribed by his pediatrician in Johannesburg. He was referred and admitted to the Pediatric Haematology and Oncology Unit, Charlotte Maxeke Johannesburg Academic Hospital (CMJAH), Johannesburg, South Africa. Initial assessment showed the patient had the signs and symptoms of significant lymphadenopathy, anemia, thrombocytopenia, and neutropenia.

A bone marrow aspiration analysis showed 97% blasts, which were small to intermediate in size of high nuclear-to-cytoplasm (NC) ratio, basophilic cytoplasm with occasional folded nuclei and vacuolated cytoplasm. Immunophenotype analysis of bone marrow sample showed 85% CD19/CD10 co-expressing cells, small to intermediate-sized cells expressing the following antigens: CD45+, CD10++, CD19++, CD22+/++, HLA-DR++ and CD38+++, one half of these cells express CD13+ and one third expressed CD15 dim. Cytogenetic analysis revealed diploid karyotype with a chromosomal t(1;19) translocation. Available data characterized this case as precursor B acute lymphoblastic leukemia (ALL), with aberrant myeloid marker expression. At the time of diagnosis, the patient was one year and 11 months old. Although hepatitis B status is routinely assessed prior to commencement of chemotherapy, there was no available hepatitis B serology result on this patient. In November 2010, he was started on a Modified Berlin-Frankfurt-Munster (BFM)-95 protocol (high risk) and treatment was completed in November 2013.

In January 2014, at the age of 5 years, while on a follow up visit for his ALL to the Pediatric Hematology and Oncology Unit, CMJAH, the child was first detected to have asymptomatic HBV infection, without clinical signs of acute hepatitis. Even though the boy had received the complete HBV vaccination schedule at 7, 11 and 24 weeks after birth, he tested positive for HBsAg. The laboratory panel showed normal alkaline phosphatase, normal total bilirubin and conjugated bilirubin, normal aspartate aminotransferase (AST), mildly elevated, alanine aminotransferase (ALT), negative for anti-HBs and anti-HBc, positive for HBeAg and HBeAb. The decision was to monitor the patient, with regular assessment.

In late 2013, the mother of the child had been diagnosed with acute HBV infection and resolved the infection without treatment. Immediately after the mother’s diagnosis, the other family members were screened for HBV infection. The father tested negative for HBsAg and anti-HBs but was positive for anti-HBc. The younger female sibling of the boy tested negative for HBV infection.

In February 2015, during a follow-up, a high HBV viral load (1.7 × 10^8^ IU/mL) was detected. Further laboratory tests revealed elevated AST: 44 IU/L and ALT: 61 IU/L, with positivity for HBsAg and HBeAg, and undetectable anti-HBs. The patient was referred to the Pediatric Hepatology Unit at CMJAH and diagnosed as chronic hepatitis B (CHB) infection in the immune-tolerant phase transitioning into HBeAg-positive immune-active phase and started on oral LAM (100 mg, daily).

**Fig. 1:**
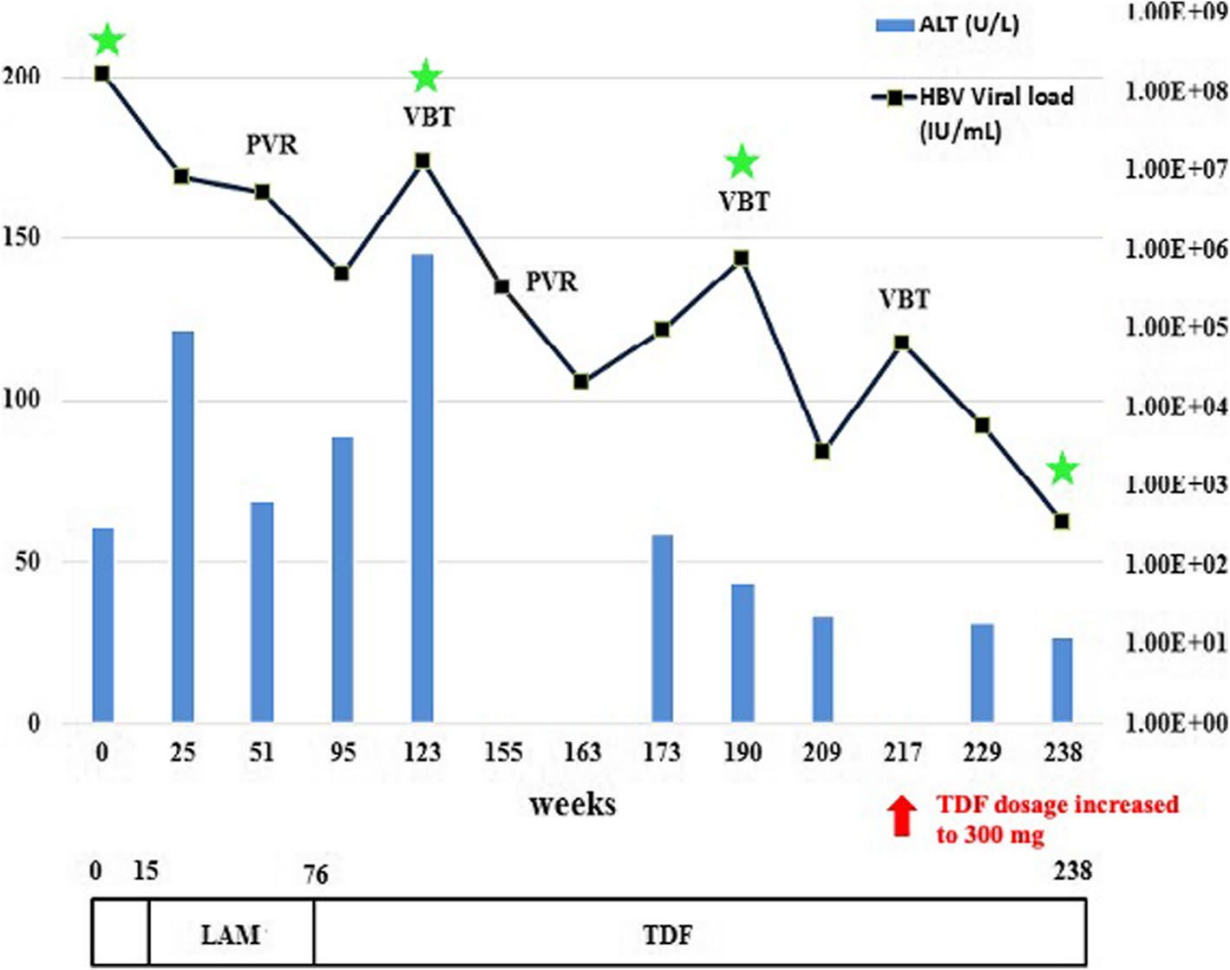
Time course of antiviral treatment and HBV viral load/ALT monitored from 2015 to 2019. The child was 23 months old when lamivudine treatment was initiated. Time points for HBeAg, HBsAg and HBsAb serology tests are shown with green stars. [Abbreviations ALT: amino alanine transferase, LAM: lamivudine, PVR: partial virologic response, TDF: tenofovir, VBT: viral breakthrough]

Upon monitoring, the first partial viral response (PVR) was detected at 36 weeks of LAM therapy (Fig. 1). PVR is defined as more than 1 log10 IU/mL decrease in HBV DNA but still detectable by real-time PCR, after at least 24 weeks of therapy of low genetic barrier (48 weeks for high genetic barrier) to resistance nucleot(s)ides analogue (NA) treatment [24, 25]. Thus, tenofovir (TDF)-based rescue monotherapy was introduced after 61 weeks of LAM therapy. The TDF dosage assessed by age and body weight was 225 mg once a day. After switching to TDF, the HBV DNA viral load continued to decline for 18 weeks (Fig. 1). Thereafter, the patient experienced viral breakthrough (VBT), at 46 weeks after the initiation of TDF. VBT is defined as ≥1 log10 IU/ml increase in serum HBV DNA level from nadir in two consecutive samples, 1 month apart, in patients who have responded and have been compliant with antiviral medication [26]. The level of ALT increased further to 146 IU/L, at the VBT event (Fig. 1).

Hepatitis D virus (HDV) is known to affect HBV viral load. To preclude the possibility of HDV/HBV co-infection or superinfection, the HDV RNA PCR test was ordered after 78 weeks of TDF treatment (National Health Laboratory Services, South Africa). The patient was HDV-negative.

Concurrently, HBV DNA was extracted from serum, using the QIA Amp DNA mini kit (Qiagen, Hilden, Germany) according to the manufacturer’s instructions. The complete full-length HBV genome was amplified using the methods described previously, with the Platinum™ SuperFi™ PCR master mix (Invitrogen, Carlsbad, CA, USA) after 78 weeks of TDF treatment [27]. PCR products were directly sequenced using the Sanger protocol (Inqaba Biotech, Pretoria, South Africa). Assembly of the sequenced fragments was performed using an in-house developed bioinformatic tool [28] and the generated full length consensus sequence blasted on the NCBI database (https://www.ncbi.nlm.nih.gov/). The complete nucleotide sequence of HBV isolate has been deposited in GenBank under the accession number OM256457. The alignment of the sequenced full-length HBV to representative full-length genotype A to E sequences available in the public repository was performed by MUSCLE v3.8 algorithm (Edgar Robert C). Phylogenetic analysis with bootstrap evaluation was performed using the maximum likelihood method with the Gamma Generalized Time Reversible (GRT + G) model of nucleotide substitution as implemented in the RAxML version 8.0.20 [29]. The resulting phylogenetic tree was converted to midpoint rooted, annotated and visualized using the Interactive Tree of Life version 5.7 [30].

**Fig. 2:**
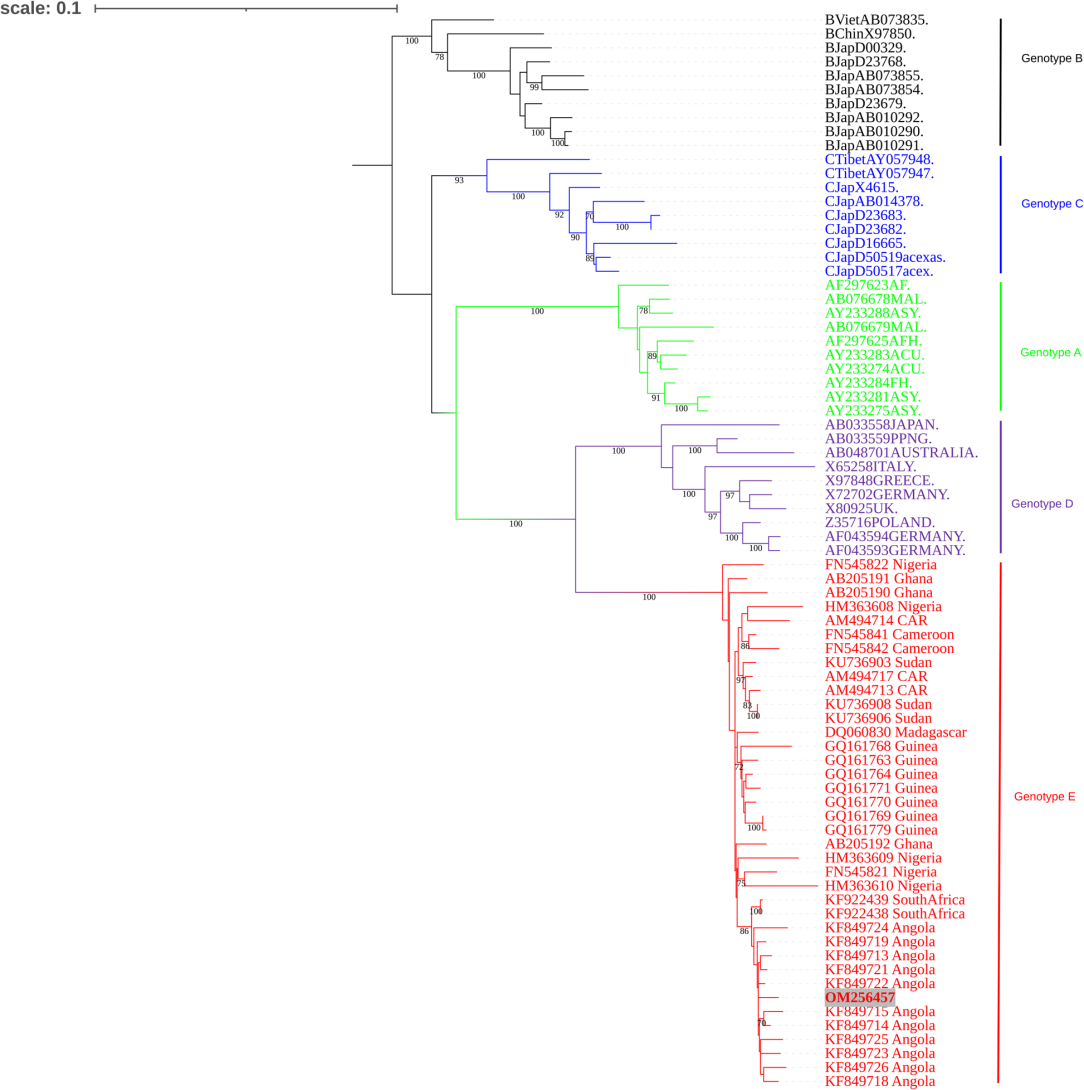
Phylogenetic analysis of HBV full-length sequence isolated from paediatric patient. A midpoint rooted phylogenetic tree constructed by maximum likelihood using RaxML is supported by bootstrap values performed at 1000 replicates with a cut off value of 80%. The reference strains from GenBank are designated by their accession number and the country of origin/isolation. The tree is rooted to an outlier sequence of genotype B from Vietnam. The sequenced isolate OM256457 is shown in red with a grey background

Phylogenetic analysis of full-length HBV genome revealed the patient was infected with genotype E, clustering with reference sequences from Angola (Fig. 2). When compared to the consensus genotype E sequence, LAM but no TDF resistance mutations were detected. Analysis from deduced amino acid sequences showed the presence of specific substitutions. Most of the mutations were localized to HLA class I- and II-restricted epitopes, CD4^+^ T-, CD8^+^ T-, and B-cells immune epitopes (Table 1). With the absence of TDF mutation, the decision was to continue with TDF-treatment. TDF dosage was further adjusted to 300 mg after 141 weeks of TDF rescue-therapy when body weight was above 35 kg. Between 113 to 141 weeks of TDF rescue-therapy, another two events of VBT were observed, with the overall trend of decreasing serum HBV DNA, and progressively declining serum AST and ALT activities. However, the patient remained HBeAg-positive, HBsAg-positive and HBsAb-negative throughout his drug therapy (Fig. 1).

**Table 1:** HBV mutations identified from the pediatric patient after 78 weeks of TDF rescue treatment

HBV Genomic Region	Nucleotide Position	Amino acid Position	Immune epitope region or nucleos(t)ide resistance
***PreS1***	T3138G	N97K	B-cell epitope residues 94–117 CD4+ T-cell epitope residues 94–108
***PreS2***	Wild-type with C9T mix	Wild-type with T7I mix	B-cell epitope residues 3–15 HLA class I/II-restricted epitope residues 1–15
***PreS2***	Wild-type with G20 A mix	Wild-type with A11T mix	B-cell epitope residues 3–15 HLA class I/II-restricted epitope residues 1–15
***PreS2***	Wild-type with C144T mix	Wild-type with P52L mix	Translocation motif (TLM) residues 41–52 HLA class I-restricted epitope residues 44–53
***S***	C324T	T57I	HLA class II-restricted epitope residues 54–69
***X***	G1461C	V30L	B-cell epitope residues 29–48
***X***	A1480G	D36G	B-cell epitope residues 29–48
***X***	A1511G	A47T	B-cell epitope residues 29–48
***Core***	A2092T	E64D	CD4 + T-cell epitope HLA class II-restricted epitope residues 50–69
***Core***	Wild-type with T2198A mix	Wild-type with L100I mix	CD8 + T-cell epitope residues 84–101 HLA class II-restricted epitope residues 81–105
***Polymerase-spacer***	G3039T	G68W	B-cell epitope residues 225–250
***Polymerase- reverse transcriptase***	A895G	S256G	LAM resistance
***Polymerase- reverse transcriptase***	A928T	M267L	LAM resistance
***Polymerase- reverse transcriptase***	C1060A	L311I	HLA class I-restricted epitope residues 306–314

The HDV RNA PCR test was repeated when the low viral load persisted after 141 weeks of TDF treatment. The second HDV PCR test was also negative. In view of the recognised TDF associated nephrotoxicity, renal function was regularly monitored by use of urinalysis, serum urea & electrolytes, serum phosphorus, magnesium, calcium, creatinine and calculation of estimated glomerular filtration rate (eGFR) [31]. Values of all these parameters were normal throughout the patient’s follow-up and will continue to be monitored while the patient remains on TDF. Since prolonged low HBV viremia is associated with a higher risk of HCC, the patient will be continuously monitored until the treatment target is reached.

## Discussion and conclusion

The infection of the child, even though he had been vaccinated against HBV, was possible, especially considering his mother was HBV-positive, the child was diagnosed with ALL, had received a blood transfusion and was treated with the Berlin-Frankfurt-Munich (BFM)-95 regimen. Furthermore, mutations in the HBV isolated may be responsible for persistence of the virus and immune escape despite treatment with LAM and TDF.

Phylogenetic analysis revealed the child was infected with genotype E HBV, which is prevalent in West and Central Africa [32]. All genotype E strains have the same characteristics: an in-frame deletion of three nucleotides (one amino acid) in the 5′-pre-S1, a signature pattern of amino acids in the pre-S1 region and the serological subtype *ayw*4. Another unique attribute of genotype E strains is the introduction of another start codon Met83 in the pre-S1 region that may result in the translation of an elongated middle hepatitis B surface protein [33]. Moreover, the HBV sequence clustered with sequences of the southwest African origin (Angola, Namibia, and DRC), which have unique mutations of T57I in the s gene and V30L in the *x* gene [29, 34].

From the child’s history, two routes of transmission can be implicated: either mother-to-child transmission, considering the mother was diagnosed with CHB or transmitted by the blood transfusion received in Angola, before the diagnosis of ALL. There is a possibility that the child infected the mother. As the mother had spontaneously resolved the infection, we were unable to isolate and sequence HBV from her blood, to determine the infecting genotype. HBV seroprevalence among blood donors in Angola has been reported to be relatively high at 6.74% [35] and thus the blood transfusion may be a possible source of the infection. In most sub-Saharan African countries, including Angola, quality assurance of screening transfusion transmissible infections remains a key challenge to ensure blood safety. In countries with resource limitations, rapid diagnostic tests with lower sensitivity and specificity are extensively used, including hepatitis B core antibody testing (anti-HBc) at the exclusion of more sensitive HBV nucleic acid testing [36]. Furthermore, the strain of HBV isolated from the child was found to co-cluster with HBV isolated previously from Angola and to share signature amino acids of that clade (Fig. 2) [29, 30].

Treatment for ALL ended in November 2013 and HBV infection and its clinical manifestations, including ALT elevations (Fig. 1), were evident 16 months later, when the immune reconstitution would have commenced. ALL is known to be immunosuppressive and 52% of pediatric hematologic malignancy patients lose immunity against HBV [37]. Moreover, anti-neoplastic treatments suppress cellular and humoral immunity in children with ALL during chemotherapy and long term remission [38–40]. The majority of Korean pediatric ALL patients (78%) lost anti-HBs after chemotherapy [41]. The BFM-95 protocol administered is immunosuppressive, with methotrexate suppressing the B-cell compartment [42], and cyclophosphamide and cytarabine depleting early linkage T-cells, and affecting T-cell proliferation [43]. From this case it is evident that even though the child had been vaccinated, it is important for practicing oncologists in HBV endemic areas to actively screen pre-chemotherapy patients for HBV, administer antiviral prophylaxis to reduce the incidence of reactivation or infection [44]. Reactivation of HBV infection is a well-described complication associated with hematologic disorder patients undergoing chemotherapy treatment. Viral reactivation can arise in post-initiation, between the treatment cycle and post-therapy [45]. Even during the immune reconstitution period, ALL children in remission have defective humoral and cellular immunity and are more predisposed to acquire severe infection [46–48]. Younger children experience a longer period of immune suppression and prominent delayed B-cell recovery [49–51]. This leads to vaccine-acquired immunological memory loss, diminished immunoglobulin titers to a level below protection and restricted immune functions [52].

To achieve persistent infection, HBV exploits different strategies to evade the host’s adaptive immunity by targeting the CD4^+^ T-, CD8^+^ T-, regulatory T-, and B-cells [53]. A weaker CD4^+^ T-cell response will promote the accumulation of a mutant strain, immune escape, and prolong persistence. Amino acid substitutions, which are significantly associated with liver disease progression from the inactive carrier through CHB to cirrhosis and/or HCC [54], were identified in the B-cell, T-cell, HLA class I, and II-restricted epitopes of HBV (Table 1). These substitutions can affect the binding affinity and interaction of antigenic peptides to both the presenting HLA epitopes and T-cell receptors, altering immune recognition, and affecting priming, activation, and proliferation of HBV specific CD4^+^ T-cells [55] and CD^+^ 8 T-cells [56]. This will affect B-cell activation, clonal expansion, and differentiation, decreasing neutralizing immunoglobulin production and delaying viral clearance.

*preS1*N97K is located within the overlapping B-cell /T-cell epitope region [57, 58], which is frequently mutated in HBV from CHB patients [54]. Similar to preS1, the preS2 also encompasses immune-dominant B- and T-cell epitopes [59, 60]. In particular, preS2 residues 6–8 (STT) are essential to maintain stable antigenic structure in this region [61]. Thus, the *preS2*T7I can affect the hydrophilicity and reduce antigenicity. *s*P52L is inside the HLA class I-restricted epitope of the HBsAg [62]. It was previously found in HBV isolated from immunosuppressed post-liver transplant patients with subsequent recurrence of HBV infection, following anti-HBs immunoglobulin treatment [63], from a HBsAg-positive HCC patient [64] and from vaccinated children in Taiwan [65]. Three mutations, namely *x*V30L, *x*D36G, and *x*A47T mapped to the B-cell epitope of HBx protein (residue 29–48) [66]. These mutations are frequently found in HBV isolated from HCC patients [54]. Residue 64 in the core gene maps to a well-characterized main immune dominant CD4 + T-cell epitope [67]. *c*E64D was previously reported to reduce T-cell proliferation in vitro [55] and correlated to HBV-associated liver disease progression [68]. The *c*L100I mutation is found within the overlapping CD4^+^ T- and CD8^+^ T-cell epitope of core gene. This mutation is believed to develop under selection pressure from the T-cells to escape the immune clearance [69].

The residual viremia observed at 36 weeks (~ 8 months) after initiation of LAM, was indicative of PVR (Fig. 1). This inadequate response, a good predictor for VBT [70], is a result of the development of resistance mutations in response to selective pressure [71]. In fact, in addition to the immune escape mutations, two lesser-known LAM resistance mutations (*rt*S256G and *rt*M267L) developed (Table 1), which were previously identified in LAM-failed CHB patients [72]. Their spatial proximity close to the active site of reverse transcriptase (RT) domain may be responsible for the development of LAM resistance [72]. Within one year of LAM treatment, 34 to 65% of children treated with LAM can develop LAM resistance mutations [73, 74] and 25.9% VBT [75]. However, because of its low cost, safety and ease of oral administration compared to interferon-α therapy, LAM may be the only choice for first-line hepatitis B therapy for children younger than 12 years in developing countries [76]. In this case, close monitoring is advised.

TDF has the highest antiviral activity and resistance barrier of all available NA treatments for HBV [77]. It is safe, effective and well-tolerated long-term. Although no renal dysfunction was documented in our patient, TDF has a low but significant risk of kidney injury. Nephrotoxicity is characterised by a proximal renal tubular dysfunction resembling Fanconi syndrome with hypophosphatemia, hypouricemia, aminoaciduria and glycosuria. TDF nephrotoxicity can manifest as a decrease in GFR and may be associated with acute kidney injury or chronic kidney injury. Regular monitoring of proximal tubular function and serum creatinine clearance is required to minimize the risk [31]. TDF is used as monotherapy [78] both in adults and children, with 100% viral clearance at 96 weeks (22 months) treatment [79, 80]. However, in the present case a reasonable response occurred only after two VBT events at 113 weeks (26 months) and 141 weeks (32 months) of TDF treatment, respectively (Fig. 1). This response is longer than that observed in genotype E HBeAg-positive infected individuals, who achieved undetectable viral load in 52 weeks (12 months), 100% response rate in 15–18 months therapy, 85.7% ALT normalized in 12 months and 100% ALT normalized in 18 months [81]. However, PVR has been documented previously, where 18% HBeAg-positive patients showed PVR after 48 weeks (11 months) TDF treatment [82]. There are a number of reasons that can account for PVR and VBT to TDF. One controversial study reported VBT associated with TDF resistance mutations [83]. However, this was not the case in the present study because no TDF mutations were detected (Table 1). It is possible that the high viral loads before initiation and at 12 weeks (3 months) of treatment may be responsible. Pre-treatment HBeAg positivity, high baseline HBV DNA (viral load ≥6.34 log 10 IU/mL), and 3rd month (viral load ≥1.91 log 10 IU/mL) are recognized as predictive factors for non-response during TDF therapy [82]. The observed multiple incidences of VBT and PVR in this pediatric patient can be attributed to the underlying delay/partial immune reconstitution from chemotherapy for ALL and the presence of immune escape mutations described above. However, the most common TDF VBT is related to non-compliance [78]. Unfortunately, the test for compliance is not available in South Africa to preclude this. As a precautionary measure, parents of the patient were educated on the importance of therapy compliance.

This case report describes an ALL paediatric patient, who developed genotype E CHB and when treated with LAM followed by TDF, showed PVR and VBT. Although TDF is an effective rescue therapy for patients who do not respond to LAM, it is important that the treatment is prolonged, especially in immunocompromised cases, in order to achieve the desired response of viral clearance. Moreover, it is important that children initiating chemotherapy for ALL are screened for immunization against HBV, even if they had previously been vaccinated for HBV infection, especially in endemic areas.

## Data Availability

All the information supporting our conclusions and relevant references are included in the manuscript. The dataset generated and/or analysed during the current study is available in the GenBank repository https://www.ncbi.nlm.nih.gov/genbank/ [Accesssion number: OM256457].

## Abbreviations

ALT: Alanine aminotransferase
ALL: Acute lymphoblastic leukemia
AST: Serum aspartate aminotransferase
BFM: Berlin-Frankfurt-Munich
CHB: Chronic Hepatitis B
HBV: Hepatitis B virus
HDV: Hepatitis D virus
HCC: Hepatocellular carcinoma
LAM: Lamivudine
NA: Nucleot(s)ides analogue (NA)
PVR: Partial virologic response
TDF: Tenofovir disoproxil fumarate
VBT: Virologic breakthrough
